# Additive Manufacturing of Nanocellulose Aerogels with Structure‐Oriented Thermal, Mechanical, and Biological Properties

**DOI:** 10.1002/advs.202307921

**Published:** 2024-03-13

**Authors:** Deeptanshu Sivaraman, Yannick Nagel, Gilberto Siqueira, Parth Chansoria, Jonathan Avaro, Antonia Neels, Gustav Nyström, Zhaoxia Sun, Jing Wang, Zhengyuan Pan, Ana Iglesias‐Mejuto, Inés Ardao, Carlos A. García‐González, Mengmeng Li, Tingting Wu, Marco Lattuada, Wim J. Malfait, Shanyu Zhao

**Affiliations:** ^1^ Laboratory for Building Energy Materials and Components Swiss Federal Laboratories for Materials Science and Technology, Empa Dübendorf 8600 Switzerland; ^2^ Department of Chemistry University of Fribourg Fribourg 1700 Switzerland; ^3^ Cellulose and Wood Materials Laboratory Swiss Federal Laboratories for Materials Science and Technology, Empa Dübendorf 8600 Switzerland; ^4^ Department of Health Science and Technology ETH Zürich Zürich 8092 Switzerland; ^5^ Center for X‐ray Analytics Swiss Federal Laboratories for Materials Science and Technology, Empa Dübendorf 8600 Switzerland; ^6^ Institute of Environmental Engineering ETH Zürich Zürich 8092 Switzerland; ^7^ School of Light Industry and Engineering South China University of Technology Guangzhou 510641 China; ^8^ Laboratory for Advanced Analytical Technologies Swiss Federal Laboratories for Materials Science and Technology, Empa Dübendorf 8600 Switzerland; ^9^ AerogelsLab, I+D Farma Group (GI‐1645), Department of Pharmacology Pharmacy and Pharmaceutical Technology Faculty of Pharmacy iMATUS and Health Research Institute of Santiago de Compostela (IDIS) University of Santiago de Compostela Santiago de Compostela E‐15782 Spain; ^10^ BioFarma Research group Department of Pharmacology Pharmacy and Pharmaceutical Technology Innopharma Drug Screening and Pharmacogenomics Platform Centro Singular de Investigación en Medicina Molecular y Enfermedades Crónicas (CiMUS) University of Santiago de Compostela Santiago de Compostela E‐15782 Spain

**Keywords:** alignment, anisotropic, cellular viability, cellulose aerogel, thermal insulation

## Abstract

Additive manufacturing (AM) is widely recognized as a versatile tool for achieving complex geometries and customized functionalities in designed materials. However, the challenge lies in selecting an appropriate AM method that simultaneously realizes desired microstructures and macroscopic geometrical designs in a single sample. This study presents a direct ink writing method for 3D printing intricate, high‐fidelity macroscopic cellulose aerogel forms. The resulting aerogels exhibit tunable anisotropic mechanical and thermal characteristics by incorporating fibers of different length scales into the hydrogel inks. The alignment of nanofibers significantly enhances mechanical strength and thermal resistance, leading to higher thermal conductivities in the longitudinal direction (65 mW m^−1^ K^−1^) compared to the transverse direction (24 mW m^−1^ K^−1^). Moreover, the rehydration of printed cellulose aerogels for biomedical applications preserves their high surface area (≈300 m^2^ g^−1^) while significantly improving mechanical properties in the transverse direction. These printed cellulose aerogels demonstrate excellent cellular viability (>90% for NIH/3T3 fibroblasts) and exhibit robust antibacterial activity through in situ‐grown silver nanoparticles.

## Graphical abstract

Cellulose fibers of various length scales are used to ink‐write complex‐shaped aerogels directly. High‐fidelity shapes are achieved, while anisotropic mechanical and thermal properties result from cellulose nanofiber (CNF) and cellulose nanocrystal (CNC) alignment. Alignment doubles tensile strength longitudinally and increases thermal resistance transversely. The printed objects maintain structure and mechanical properties in the transverse direction through drying and rehydration cycles, which is essential for biomedical applications.

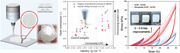

## 1 Introduction

Cellulose stands out as the most abundant biopolymer on Earth.^[^ ^1^ ^]^ It is a complex carbohydrate that provides rigidity and strength to the cell walls of plants.^[^ ^2^ ^]^ Cellulose is renewable and biodegradable and can be broken down by natural processes, unlike synthetic materials that can persist in the environment.^[^ ^1^, ^3^ ^]^ This makes cellulose an attractive option to reduce the environmental footprint. Cellulose aerogels have attracted considerable attention due to their high surface area, and they can be efficient absorbents for pollutants, oils, and other contaminants.^[^ ^4^ ^]^ Moreover, cellulose aerogels exhibit excellent mechanical strength for their low density. They can withstand large deformations without breaking, making them useful for structural applications such as lightweight composites and scaffolds for tissue engineering.^[^ ^5^ ^]^ The renewable and non‐toxic nature of cellulose aerogels also makes them environmentally friendly alternatives to synthetic polymer aerogels or inorganic silica aerogels. However, the lightweight nature of cellulose aerogels is usually mechanically weak, posing a challenge for conventional methods of producing complex shapes and geometries. To overcome this limitation, 3D printing can precisely control the final dimensions and geometries and enable the creation of complex, customized shapes and architectures that are tailor‐made for specific applications.^[^ ^6^, ^7^ ^]^ However, when considering different printing technologies, two‐photon printing (TPP) can achieve high‐resolution printing in sub‐micrometer scales but faces challenges in printing large‐sized samples. Fused deposition modeling (FDM) or selective laser sintering (SLS) can produce large‐sized samples with complex macroscopic geometries, but microstructure tuning becomes difficult.^[^ ^8^ ^]^ Recent studies have reported the successful fabrication of cellulose‐based porous materials using a direct ink writing (DIW) additive manufacturing technique.^[^ ^9^, ^10^, ^11^, ^12^, ^13^ ^]^ The shape rigidity of DIW structures depends on the visco‐elastic response and hardening method employed, which can be tuned for optimal printed structures.^[^ ^9^, ^10^, ^14^ ^]^ The processing that follows the extrusion response dictates the usability of the printed structure and ranges from simple solvent/anti‐solvent interactions to complex polymerization reactions.^[^ ^11^, ^15^ ^]^ Yet, it is still challenging but interesting to employ the DIW method to achieve high shape fidelity in large‐sized objects while fine‐tuning the microstructure and desired properties.^[^ ^10^ ^]^

**Figure 1:**
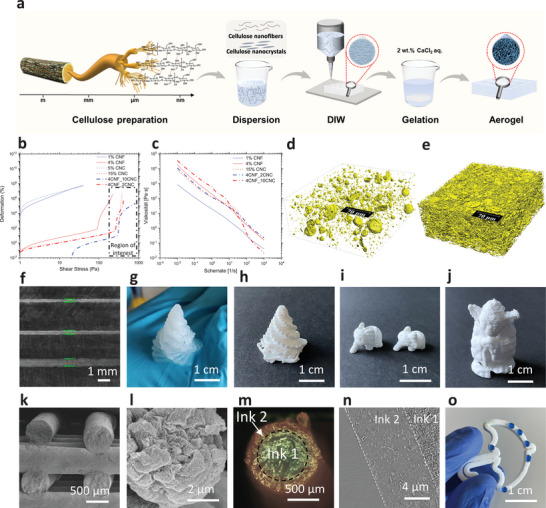
Additive manufacturing of pure nanocellulose hydrogels and aerogels. a) Process scheme: CNF and CNC extraction from wood pulp, dispersion in water, and concentration adjustment to tune the ink rheology and DIW of the cellulose inks, initiate gelation by immersion in a 2 wt.% CaCl_2_ solution, post‐processing, and supercritical CO_2_ drying. b) Amplitude sweep of inks with various CNF and CNC combinations. c) Shear‐thinning behavior. d) 3D volume rendering of a 4CNF_2CNC aerogel and, e) 4CNF_10CNC sample: higher density CNC‐CNF mixed aggregates (yellow, 1.3 vol.% for 4CNF_2CNC and 15.5 vol.% for 4CNF_10CNC) embedded in low‐density matrix (predominantly CNF) (white) (200×200×80 µm^3^, effective voxel size of 162.5 nm). f) Filaments printed from 250‐µm, 410‐µm, and 840‐µm nozzles. Printed g) hydrogel and h–j) dried gel objects. k) SEM cross‐section of a dried grid structure. l) SEM observation of the mesoporous aggregates. m) Coaxial printing of hydrogels with 4CNF_2CNC shell and 4CNF_2CNC_graphite core. n) Longitudinal µCT cross‐section of the core‐shell filament. o) 3D printed hydrophobic 4CNF_10CNC aerogel, xCNF_yCNC stands for x wt.% CNF and y wt.% CNC in the ink formulation.

We present a novel approach to DIW printing of pure cellulose aerogels by combining cellulose fibers of different length scales, i.e., CNFs suspension as the binder phase and CNCs as the filler phase (**Figure** **1**a; Table S1, Supporting Information). The printed aerogels exhibit a high faction of nanopores with remarkable anisotropic mechanical and thermal properties due to CNF and CNC alignment (quantified by Small‐angle X‐ray scattering (SAXS)). Specifically, the alignment doubles the tensile strength in the longitudinal direction, and the thermal resistance was significantly higher in the transverse direction than in the longitudinal direction (24 versus 65 mW m^−1^ K^−1^). Remarkably, the printed objects withstand cycles of drying and rehydration while maintaining good pore structure and greatly improved mechanical properties. This characteristic is crucial for biomedical applications by promoting cell viability and enabling surface modification. The biomedical potential was tested in vitro using cell growth experiments and antibacterial testing.

## 2 Results and Discussions

### 2.1 Direct Ink Writing of Cellulose Aerogels Utilizes Nanofibers of Varying Length Scales

The rheological properties of the inks were adjusted to fulfill the printing requirements (Figure 1b,c) by tuning the absolute and relative concentrations of each phase. The CNFs have a large aspect ratio (≈6.1 ± 1.0 nm in diameter, several micrometers in length),^[^ ^16^ ^]^ which leads to high viscosities even at low concentrations, e.g., a 1.5 wt.% CNF suspension has a viscosity 3000 times higher than pure water, and when the CNF concentration increases to 4 wt.% CNF, the viscosity increases by a further order of magnitude (Figure S1, Supporting Information). In theory, higher CNF concentrations can improve yield stress and printability. Still, the maximum CNF concentration is limited to 4 wt.% because of inhomogeneity and inadvertent film formation at higher concentrations. As an alternative, CNC with a lower aspect ratio (diameter 5 nm, length 150–250 nm) increases the aerogel strength and reduces the deformation of the printed filament without macroscopic inhomogeneity.^[^ ^17^ ^]^ As the CNC concentration rises from 2 to 10 wt.%, the yield stress increases (e.g., higher than 100 Pa), improving printability (Figure 1b; Figure S2, Supporting Information). Even at more moderate CNF concentrations (1–4 wt.%), the high viscosity induces the formation of 5 to 40 µm spherical CNC/CNF aggregates, which have a higher local density than the surrounding CNF binder phases (Figure 1d,e,l). These aggregates are not solid particles but have a mesoporous structure (Figure 1l). The µCT analysis of the corresponding aerogels (Figure 1d,e) indicates aggregate sizes of 5–20 µm; the interactions between these aggregates^[^ ^14^ ^]^ improve the rheological properties (high yield stress and improved shear thinning behavior) of the inks. Printing with unsuitable printing parameters (speed and pressure) or inks with unsuitable rheology can lead to swelling of the gel filaments and cause defects to propagate during the printing process (Figure S3, Supporting Information). Still, these issues can be easily avoided by optimizing the ink and print parameters to maintain high shape fidelity. The optimized CNC/CNF inks can be printed through different size nozzles (410, 600, and 800 µm, Figure 1f) to manufacture hydrogels with complex geometries, including bridging structures and overhangs (Figure 1g; Figure S4, and Movie S1, Supporting Information). The printed objects can then be post‐processed (CaCl_2_‐induced gelation, solvent exchange, and supercritical CO_2_ drying) to obtain the pure cellulose aerogel structures (Figure 1h–l). The filaments maintain their original shape (Figure 1k), and two ink formulations could be printed simultaneously to produce core‐shell structures (Figure 1m; Figure S5, Supporting Information). After drying, a smooth interface with a sharp transition between the core and shell could be identified (Figure 1n). Optionally, to improve the cellulose aerogel's stability in different usage conditions (e.g., wet atmosphere or humidity sensitivity), the printed cellulose can be hydrophobized during the post‐processing (Figure 1o).

### 2.2 Directional Arrangement of the Gel Structures and Properties

**Figure 2:**
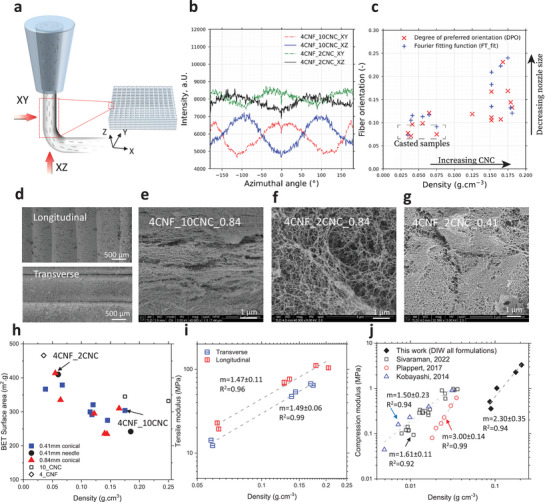
Alignment of the structure of cellulose aerogel during the printing. a) Schematic representation of the printed filament directions the potential alignment of the cellulose nanofibers. b) Radial integration of SAXS profiles for two different CNC concentrations (in the range of 0.3 < q < 1 nm^‐1^), with X (dashed lines) and Y (solid lines) filament showing a phase difference of 90°. c) Fiber orientation using DPO and FT_fit for DIW‐aerogels as a function of density. d) The printing filament directions. Microstructure of the printed objects with e) ink 4CNF_10CNC and nozzle size 0.84 mm, f) ink 4CNF_2CNC and nozzle size 0.84 mm, g) ink 4CNF_2CNC and nozzle size 0.41 mm. h) Specific surface area for different CNF‐CNC aerogels using different nozzle types. Open symbols are CNF‐ and CNC‐only samples. i) Tensile modulus of the objects tested from transverse or longitudinal directions. j) Compression modulus as a function of density for the DIW printed aerogels and a comparison with reported mold‐casted cellulose aerogels.^[^ ^19^, ^21^, ^22^ ^]^

The printed samples display anisotropic properties caused by the directional arrangement of the gel structures during the printing of structural features on different length scales (**Figure** **2**a). At the nanometer scale, the CNFs and CNCs can be aligned during extrusion,^[^ ^18^ ^]^ and this alignment can be probed by SAXS and WAXS measurements (Figure 2b; Figures S6 and S7, Supporting Information). SAXS probe electron density fluctuations over length scales between 1 and 100 nm and, in this case, are more sensitive to CNFs alignment, whereas WAXS probe the alignment of crystalline matter and is sensitive to the alignment of CNCs, with the caveat that the CNF themselves also contain signatures of the CNCs (Figure S7, Supporting Information). The azimuthal SAXS intensities (Figure 2b) and the degree of alignment calculated with the Hermans' orientation algorithm (Figure 2c) display an increasing alignment trend with increasing CNC addition. The WAXS data also display a moderate degree of orientation, even somewhat higher than the SAXS data, but with no clear dependence on ink or printing parameters (Figure S6, Supporting Information). Given the observed aggregations of CNC (Figure 1d,e), this CNC alignment's origin is unclear. In SEM observations of the overall arrangement of the fibrillar structures, there appears to be some degree of CNF orientation and organization into sheets (Figure 2d), and this effect becomes more pronounced when the CNC concentration is increased (Figure 2e,f) or the inks are extruded through a narrower nozzle (Figure 2g). Overall, the alignment is less pronounced in the printed aerogels compared to those densified by uniaxial compression^[^ ^19^ ^]^ or to the alignment of CNC in CNC‐reinforced polymers.^[^ ^20^ ^]^ The lower degree of alignment observed here in the printed cellulose aerogels is most likely related to the low concentration of CNC loading (10 wt.%) and the low viscosity of the fluid (water) compared to that of the molten polymer.^[^ ^20^ ^]^ It may also be influenced by solvent exchange and the ScCO_2_ drying process.^[^ ^15^ ^]^ This may enable the CNF to lose alignment when the shear is removed after extrusion. At a much larger length scale, the filament orientation is defined by the DIW protocol. The boundaries of the individual filaments can be identified even in fully filled objects (Figure 2d), which may impart anisotropy to the printed objects because the outer filament perimeter may be partially densified during printing and solidify more when exposed to the crosslinker during gelation, resulting in higher local density in the skin than in the inner structure.

The alignment of features from the nano‐ to millimeter scale imparts the printed aerogel objects with anisotropic mechanical properties (Figure 2i,j). The tensile modulus of the printed cellulose aerogels strongly depends on density with a power law behavior (≈ρ^m^, with m = 1.4–1.5) but is higher by ≈70%–80% for aerogel dogbone samples measured parallel to the printing direction compared to the transverse direction. The compressive E modulus (measured in the transverse direction) also scales with density with a power law behavior with a value of the exponent m = 2.3 within the range typically observed for cellulose aerogels, m = 1.5‐3.0,^[^ ^19^, ^21^, ^22^ ^]^ and the highest reported E‐modulus for cellulose aerogel (3.5 MPa) (Figure 2j). The anisotropy was not evaluated in compression. Increasing the CNC amount improves the overall mechanical properties but decreases the specific surface area. Indeed, the surface area of composite CNF‐CNC aerogels is lower than expected from a simple interpolation of the surface areas of neat CNF and CNC aerogels.^[^ ^19^, ^21^, ^22^ ^]^ In the high‐viscosity ink formulation, there is a higher likelihood of aggregate formation, and we anticipate that these aggregates have a more compact structure and a higher density (see Figure 1d,e), resulting in a lower overall surface area.

**Figure 3:**
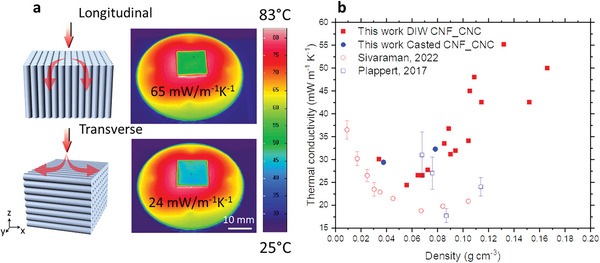
Thermal insulation properties of printed cellulose aerogels. a) Infrared images of the 4CNF_10CNC aerogel measured from longitudinal or transverse directions after 30 min on a 100 °C hotplate (emissivity of the background is set to be 1). b) Transverse thermal conductivity of printed cellulose aerogels (25 °C, 50% R.H.) and the thermal conductivities of the reported isotropic cellulose aerogels.

The alignment of structures also leads to anisotropy in the heat transport properties. When placed on a hotplate, a noticeable temperature difference is observed on the cold side depending on the printing orientation (58 vs. 43 °C) (**Figure** **3**a). Measurements confirm this anisotropic thermal conductivity (λ): 65 versus 24 mW m^−1^ K^−1^ in longitudinal versus transverse orientation (ink 4CNF_10CNC aerogel, 410 µm conical nozzle, Movie S2, Supporting Information), i.e., heat transport is 2.7 times more effective along than perpendicular to the filament direction. Thus, while the tensile properties are improved in the direction of the structure alignment, the thermal insulation performance is reduced. We cannot determine unequivocally if the anisotropy is more due to the nanoscale alignment of CNF and CNC (from the SAXS analysis) or due to mm scale density fluctuations by the alignment of the filaments and their denser structures/skin remnants. However, CNC and CNF alignment is only moderate; a much higher degree of alignment in uniaxially compressed cellulose aerogels only reduced the transverse thermal conductivity by ≈5 mW m^−1^ K^−1^ compared to isotropic aerogels.^[^ ^19^ ^]^ In addition, it is difficult to rationalize how the CNC and CNF alignment could raise the longitudinal conductivity to as high as 65 mW m^−1^ K^−1^, given the tortuosity of the networks, the small diameter of the CNC and CNF and the limited degree of alignment. Hence, we hypothesize that larger‐scale density variations, due to higher local density in the skin than in the inner filament structure, are more important.

Considering the higher thermal conductivity in the longitudinal direction, we find it pertinent to concentrate solely on the transverse measurements when analyzing the insulation properties. To test the density dependence of the transverse thermal conductivity, we tailored the CNF/CNC concentration to vary its density from 0.03 to 0.16 g cm^−3^. The data display a classic U‐shaped curve, with a minimum λ of 24 mW m^−1^ K^−1^ at a density of 0.055 g cm^−3^. This U‐shaped dependence of λ arises from competing density dependencies of radiative and gas phase versus solid phase contributions.^[^ ^23^ ^]^ The minimum λ for the printed aerogels is below the thermal conductivity of standing air (26 mW m^−1^ K^−1^), indicating that the mesoporous aerogel structure partially suppresses the gas phase conduction. However, it is higher than that of cellulose aerogels prepared by uniaxial compression (≈18 mW m^−1^ K^−1^ at a density of 0.067–0.087 g cm^−3^, Figure 3b ^[^ ^21^ ^]^), most likely because the latter aerogels are more homogeneous, i.e., they more effectively divide up the pore space into smaller pores for a given density, and because the CNF and CNC alignment is much stronger. Nevertheless, a thermal conductivity of 24 mW m^−1^ K^−1^ is substantially lower than that of conventional insulation materials or macroporous cellulose foams prepared by freeze drying, and the high anisotropy factor of 2.7 enables opportunities for lateral heat dissipation and effective heat management with a single material.

### 2.3 Rehydration‐Fortified Gel Structures and Their Biomedical Potential

**Figure 4:**
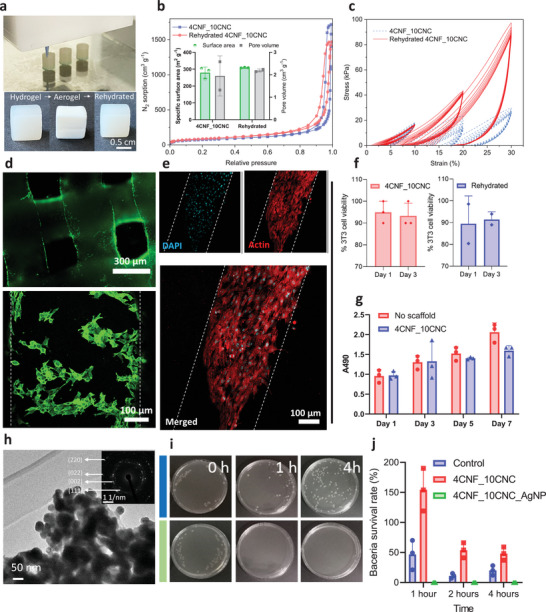
Cell culture and antimicrobial activity of the printed cellulose aerogels. a) DIW of the cylinders (diameter 6 mm, height 9 mm) for compression tests (top), and the images of the freshly printed hydrogel, dried aerogel, and rehydrated hydrogel scaffolds (ink 4CNF_10CNC) with lattice structure (grid size of ≈320 µm) for the cell culture evaluation (bottom). b) N_2_ sorption isotherms of the aerogels dried from fresh prepared and rehydrated 4CNF_10CNC and their specific surface area and pore volume (insert). c) Cyclic compression of the freshly prepared and rehydrated hydrogels from ink 4CNF_10CNC. d) NIH/3T3 cells seeded over the cellulose scaffolds (4CNF_10CNC fresh hydrogel) demonstrate high cell viability (> 90%) at day 1; green (Calcein AM) indicates viable cells, red (Ethidium homodimer) indicates dead cells. e) Confocal images of fixed samples on day 7 demonstrate that the cells proliferated over the cellulose scaffolds; blue: DAPI, red: Phalloidin (actin). f) In culture, NIH/3T3 cell viability on 4CNF_10CNC fresh and rehydrated hydrogels was high after 1 and 3 days (> 90%). g) Cellular metabolic activity (determined from the MTT assay) of cells cultured in the presence of the fresh cellulose hydrogel was not significantly different from the controls (no scaffold) during 1, 3, 5, and 7 days of culture. h) TEM image of the AgNP‐loaded cellulose aerogel and the SAED pattern of the Ag NP (insert). i) Growth of *E. coli* (OD600 = 0.3) in an LB‐agar plate with AgNP loaded 4CNF_10CNC. j) Bacteria survival rate on the AgNP loaded 4CNF_2CNC after 1, 2, and 4 h.

The printing technology developed here can precisely control the shape and geometry of cellulose aerogels (Figure 1; Movie S3, Supporting Information) and the directional dependence of pore structure and fiber alignment (Figure 2; Movie S4, Supporting Information). Given the tremendous interest in cellulose‐based materials for tissue engineering and regeneration,^[^ ^24^ ^]^ drug delivery, hemostasis,^[^ ^25^ ^]^ and implantable devices,^[^ ^26^ ^]^ our porous biopolymer 3D printable cellulose‐based substrates are viable candidates for various biotechnological or pharmaceutical applications. Specific opportunities include biomaterial implants for cartilage, skin, and bone repair and regeneration^[^ ^27^ ^]^ as biocompatible insulation or structural material for implantable electronics.^[^ ^28^ ^]^ Utilizing CO_2_‐sterilized, lightweight aerogels for storage and transportation is appealing.^[^ ^29^ ^]^ Still, a rehydration process cannot be avoided when using aerogels for biomedical applications, e.g., immersion in aqueous solutions or contact with bodily fluids. Surprisingly, the rehydration of the printed cellulose aerogels did not cause significant alteration in the pore structure (**Figure** **4**a,b). Yet, the mechanical properties were substantially better for rehydrated aerogels compared to as‐prepared hydrogels, particularly in cyclic compression (Figure 4c). This improvement may be attributed to a reinforcement, e.g., by forming additional nanofiber contacts, during the CO_2_ immersion, which is known to induce gelation^[^ ^30^ ^]^ and affect the structure formation of biopolymer gels.^[^ ^30^ ^]^ Rehydrated aerogels thus offer enhanced mechanical properties compared to the initial hydrogel state.

To determine the biocompatibility of the rehydrated aerogels, we conducted in vitro evaluation tests on NIH/3T3 fibroblasts (cell line). Here, 3D‐printed as‐prepared cellulose hydrogels allowed cell attachment (owing to the high hydrophilicity and hierarchical pore structure) and demonstrated no adverse effect or interference on cellular viability and metabolic activity (Figure 4d–g, Figure S8, Supporting Information). Rehydrated aerogels showed comparable cell viability to the as‐prepared hydrogels (Figure 4f). Thus, rehydrated cellulose aerogels warrant future exploration for specific biomedical applications as implantable materials or in combination with other bioactive materials such as collagen, chitosan, or alginate,^[^ ^31^ ^]^ to investigate the effects on gene expression (proliferative, pro‐inflammatory, etc.) on the primary mouse or human cells (e.g., macrophages, fibroblasts, and mesenchymal stem cells).

Antimicrobial applications are another area of interest for 3D‐printed cellulose‐based materials. Cellulose hydrogels without antimicrobial agents promote bacterial proliferation (Figure 4g), which may limit their practical use. The addition of silver nanoparticles (Ag‐NPs) during printing was shown to be an effective means of suppressing bacterial growth on cellulose substrates.^[^ ^32^ ^]^ As a proof‐of‐concept, Ag‐NPs were incorporated into 3D printed cellulose aerogels through in situ synthesis (Figure S9a, Supporting Information) as evidenced by the strong diffraction peaks at 2θ = 38.16° (111), 47.33° (101), 58.91° (105), and 64.88° (110) of the face‐centered‐cubic (FCC) structure of Ag (Figure 4h). The in‐situ generated Ag‐NPs are distributed homogeneously (Figure S9b, Supporting Information) and effectively prevent bacterial infections in cell cultures (Figure 4i,j; Figure S9c,d, Supporting Information). This composite can be particularly useful in applications such as tissue engineering or implantable medical devices, where bacterial infection leads to severe complications.

## 3 Conclusion

In summary, the combined CNC and CNF inks demonstrate superior, stable, and consistent printability for direct ink writing of anisotropic, pure cellulose hydrogels and aerogels. The water‐based inks and convenient Ca^2+^‐induced gelation open a broad printing window for large and complex geometries. The structure alignment during extrusion endows the printed aerogels with unique anisotropic properties and exceptional thermal and mechanical properties, making practical applications feasible under demanding conditions. The large specific surface area offers ample opportunity to add functional groups or nanoparticles, as shown here with antimicrobial silver nanoparticles. The 3D printing technology is a versatile and scalable approach to fabricating cellulose aerogels with tailored properties and geometries for a wide range of applications, especially in thermal insulation and biomedical fields.

## 4 Experimental Section

### Materials Sourcing

Cellulose nanocrystals (CNC) from sulfuric acid hydrolysis of eucalyptus pulp were produced at USDA Forest Service‐ Forest Products Laboratory (Madison, WI, USA) and were purchased from the University of Maine as freeze‐dried powder. Never‐dried elemental chlorine‐free (ECF) cellulose fibers from bleached softwood pulp (*Picea abies* and *Pinus spp*.) were acquired from Stendal GmbH (Berlin, Germany). 2,2,6,6‐Tetramethyl‐1‐piperidinyloxyl (TEMPO) and sodium hypochlorite (NaClO) solution (12%−14% chlorine) were procured from VWR international. Sodium bromide (NaBr ≥ 99%) and sodium hydroxide (NaOH ≥ 99%) were supplied by Carl Roth GmbH. 95/5 wt.% Ethanol/IPA was purchased from Alcosuisse AG. Calcium chloride (CaCl_2_) was purchased from Sigma–Aldrich GmbH.

### TEMPO‐Oxidized CNF Preparation

*CNF processing was conducted, as discussed in the* literature.^[^ ^33^, ^34^, ^35^, ^36^ ^]^ The TEMPO‐oxidized CNF received from Masuko processing was ≈1.2 ± 0.05 wt.%, which was subsequently homogenized and diluted for 10 passes till a uniform transparent suspension was reached (M110EF, Microfluidics Ind., Newton, MA, USA; 0.7 ± 0.03 wt.%). This suspension was ultrasonicated (Branson Digital horn sonicator) for 10 mins at 45% amplitude. This suspension was further concentrated using a vacuum distillation (Rotavap, BÜCHI Labortechnik AG, Switzerland) to 1–4 wt.% per desired final CNF concentration.

### Ink Preparation

Freeze‐dried CNC powders were added as is, according to a dry weight ratio of 1–10 wt.% to different concentrations of CNF, along with 2 ceramic balls (Φ = 1 cm). They were thoroughly mixed (SpeedMixer DAC 150, 1 FVZ) at 1000 and 1500 rpm for 1 and 2 min, respectively. The balls helped break down the coarse agglomerates, but some large agglomerates were manually crushed again, after which the ink was remixed at 1500 and 2300 rpm for 1 and 2 mins, respectively. These inks were stored overnight at 4 °C to facilitate swelling of the inks and were pre‐mixed again at 2300 rpm for 1 min, after which they were loaded into plastic syringe cartridges (Adhesive Dispensing Ltd.) and centrifuged (to remove air bubbles/gaps).

### 3D Printing

CNF‐CNC inks were printed using a direct ink writing (DIW) procedure^[^ ^20^ ^]^ using EnvisionTEC (Bioplotter Manufacturer Series, USA). Depending on the ink rheology, hydrogels were extruded through different HDPE (tapered conical) and stainless‐steel nozzles (tapered needle) of different diameters (1.2 to 0.41 µm) using different air pressures (0.2–3.0 bar), translational speeds (10 – 30 mm·s) at 10 °C. The bed temperature was kept at 10 °C to avoid drying during printing. The model for printing (45×45×6 mm for λ measurements) was made using Autodesk Inventor (Autodesk Inc., USA) or taken from the Thingiverse repository (links mentioned in Supporting Information). For anisotropic orientation, samples were printed at 90° to the original model (45×6 mm, 45 mm high). The extruded hydrogels were printed on a glass substrate to avoid surface adhesion or reaction. After printing, the samples were immediately placed in a 2 wt.% CaCl_2_ gelation bath for 10 min (this crosslinking is only possible due to the carboxylic groups on the TEMPO‐oxidized CNF used in the ink composition), after which a spatula was used to carefully remove the hydrogel from the substrate into a container with 35 vol.% ethanol.

### Hydrophobization

The modification process occurred in a 1 L tall, closed reaction vessel (VWR number: 0 600 0270, suitable for 45×45×10 samples) equipped with an electrical heating jacket for precise temperature control. In this setup, 20 mL of organogel was immersed in 150 mL of the exchanged solvent within the vessel. The system was then purged with N_2_ for 15 mins to remove residual O_2_. Subsequently, the vessel was heated to 80 °C, and a three‐molar excess of acid chloride was added (1.44 mL C6, 2.164 mL C12, 2.54 g C18) based on the molar ratio of acid chloride to total OH groups (3:1). The reaction proceeded for 7 h. A 3‐necked round bottom flask was used for smaller samples, yielding similar results. Increasing the reaction temperature can reduce the reaction time; however, the vapor pressure within the enclosed vessel limits the maximum temperature. After completion of the reaction, the gel was washed twice with acetone and once with the corresponding solvent to remove any excess or unreacted acid chloride. Subsequently, the gel was solvent exchanged back to heptane before undergoing supercritical drying, using the same conditions employed for hydrophilic aerogels. It's worth mentioning that samples in which the reactions were carried out in heptane did not require the intermediate acetone wash.

### Sc‐CO_2_ Drying

The hydrogel was solvent exchanged in 35‐70‐90‐99.5% ethanol steps; several steps were taken during the solvent exchange with ethanol to minimize tensile forces in the matrix due to Hansen solubility parameters and Ostwald ripening.^[^ ^37^ ^]^ These alcogels were supercritically dried using a continuous drying apparatus (Separex, Champigneulles, France) in a 500 mL autoclave for 6 h, 120 bar, 50 °C and 15 g min^−1^.

### Ag‐NPs Loaded Aerogels

DIW printed cellulose objects were immersed in the chitosan solution (>400 mPa s, 1% in acetic acid(20 °C), 85% degree of deacetylation, Sigma–Aldrich), and then the gels were solidified by ethanol, the silver ions were loaded by immersed the gels into 0.5 M AgNO_3_ water solution for 12 h, the gels were then solvent exchanged with ethanol. After Sc‐CO_2_ drying, the composites were exposed to UV irradiation (95% at 254 nm) for 10 h to form the Ag nanoparticles in situ.^[^ ^38^ ^]^

### Rehydration of the Aerogels

To check the structure change and cell viability of the aerogels after rehydration, the Sc‐CO_2_ dried aerogels were placed into a 2 wt.% CaCl_2_ solution for 12 h and cyclic compression was placed on the rehydrated aerogels. The gels were exchanged with ethanol (95/5 wt.% ethanol/IPA, Alcosuisse AG), and the alcogels were supercritically dried using a continuous drying apparatus; the dried gels were examined for pore structures using N_2_ sorption.

### Density Analysis

Monolithic samples were used for density measurement; therefore, a weighing scale and calipers were used to measure envelope density. One should note that the actual density is usually slightly lower than this value due to the small fraction of voids present due to the printing procedure. The skeletal density of cellulose aerogels was 1.6 g cm^−3^.^[^ ^39^ ^]^

### Rheology

2 mL of each sample was placed between the stationary plate and the shearing plate of a rheometer (50 mm; Anton Paar MCR 502). They were measured with a gap of 1 mm at a constant temperature of 20 °C. Yield stress measurements were done under rotation of the shearing plate between 0.01 and 1000 Pa for all samples. The flow curve (viscosity) measurements were done between 10 and 300 s^−1^ shear rates and were time‐controlled for 4.5 s between each point.

### N_2_ Sorption

Nitrogen adsorption‐desorption measurements were conducted on a Micromeritics 3flex device in a process similar to that of cellulose aerogels described in the literature.^[^ ^40^ ^]^ The specific surface area using the BET model (S_BET_; m^2^ g^−1^) and the pore volume using the BJH model (V_BJH_; cm^3^ g^−1^) were derived from the isotherms. The V_BJH_ underestimates the average pore size as we also have macropores, and our 3D‐printed samples add further micrometer‐level defects. Therefore, V_pore_ and D_pore_ were also calculated from the envelope, skeletal density, and surface area. D_pore_ selects a pore geometry because the actual pore geometry is not trivial. Estimating such an average would require an assumption (for a high‐porosity, open‐porous nanofibrous network like cellulose^[^ ^19^ ^]^). Hence, we assume a hexagonal pore arrangement as natural biopolymer fibrillar networks tend to orient themselves in maximal surface‐to‐volume polygons, e.g., natural fiber schemas and freeze‐dried aerogels. This is a better assumption than cylindrical pore geometry.^[^ ^41^, ^42^ ^]^

(1) $$V_{pore}=\;\frac{1}{\rho_{envelope}}-\frac{1}{\rho_{skeletal}}$$

(2) $$D_{pore}=\frac{4*V_{pore}}{\sqrt{3}*S_{BET}}\;$$

### Electron Microscopy

Scanning electron microscopy (SEM) images were obtained using a FEI Nova NanoSEM 230 instrument (FEI, Hillsboro, OR USA) at an accelerating voltage of 5 kV, a spot size of 2.0 and a working distance of 4 mm. A platinum coating of 10 nm was applied to avoid charging during image acquisition. Sample images were compiled using Image J. Transmission electron microscopy (TEM) images were recorded with a JEOL‐2200FS microscope operated at an accelerating voltage of 200 kV.

### Mechanical Tests – Aerogels

Uniaxial compression tests were performed on cuboidal samples of different filling angles and orientations. They were made plane parallel by sanding the final aerogels and analyzed using a universal materials testing machine (Zwick/Z010, Zwick/Roell, Germany) equipped with a 100 N force transducer cell (KAP‐S, AST Gruppe GmbH, Germany) in a controlled environment (23 °C; 50 ± 5% RH). The compression rate was kept at the following ASTM‐C109. The elastic moduli were calculated from the slope of the curve's initial linear phase (3%–5% strain). Tensile testing was done on different orientations and concentrations using the “dog‐bone” structure, using the ASTM‐D3574 norms (0.2%–0.5%).

### Mechanical Tests – Hydrogels

Cyclic loading–unloading hysteresis experiments were performed to evaluate the reversible behavior of printed hydrogels and rehydrated aerogels. The test was initiated when the 0.1 N force was reached, and 10 continuously repeated cycles were performed. The Zwick/Z010 was a 100 N force transducer cell (KAP‐S, AST Gruppe GmbH, Germany) in a controlled environment (temperature = 23 °C; relative humidity = 50 ± 5%). The loading‐unloading test speed was 2 mm min^−1^.

### Thermal Conductivity

Thermal conductivities (λ) were measured with a miniaturized ISO_12 667/European Standard EN12667^[^ ^43^ ^]^ guarded hot‐plate device (with shielding in the XY plane), calibrated from industry standard measurements for an expanded polystyrene sample of 500×500mm^2^. The printed samples were equilibrated in the room's atmosphere where the device was located (23 °C, ≈40% RH) overnight before measurements. The temperature of the cold plate was ≈20 °C (uncontrolled), and the hot plate was maintained at 30 °C (to mimic the ISO standard). Each sample was measured at steady–state heat flow (≈1 h) to calculate λ after correction factors.^[^ ^44^ ^]^

### Small and Wide‐Angle X‐ray Scattering (SAXS, WAXS)

SAXS experiments were carried out on a benchtop Bruker Nanostar (Bruker AXS GmbH, Karlsruhe, Germany) using the K_α_‐line of a micro‐focused X‐ray Cu source with a wavelength of 1.5406 Å. The beam was collimated using a 0.3 mm pinhole, leading to a beam diameter of ≈0.4 mm at the sample position. The sample‐detector distance was set to 107 cm and further calibrated with silver behenate, achieving a resolvable q‐range of 0.07 ≤ *q* ≤ 2.3 nm^−1^. WAXS measurements were recorded on a similar setup with a sample‐to‐detector distance of 5 cm, further calibrated using a corundum powder sample covering a q‐range of 4.5 ≤ *q* ≤ 31 nm^−1^. The scattering vector *q* is defined as (|q|)^→^ = 4π sin2θ/λ with 2θ as the scattering angle and λ the wavelength of the X‐ray source, was recorded on a gaseous avalanche‐based detector (VÅNTEC‐2000, Bruker AXS) with 2048×2048 pixels and a pixel size of 68×68 µm^2^. The scattering patterns were recorded at room temperature under moderate vacuum conditions (10^−2^ mbar) to limit air scattering. Scattering was recorded for 30 s for each sample. The intensity of the semi‐transparent beamstop from empty beam scans was used for transmission calibration. The scattered intensity was extracted, azimuthally averaged, and integrated over each q‐value using the Bruker software DIFFRAC.EVA (Bruker AXS, version 4.1). The 1D data was transmission corrected and background‐subtracted from the scattering of the respective solvent, polymers, and the empty quartz capillary using an in‐house data pipeline operating under Matlab 2022. Integration was carried out along azimuthal angle and radial coordinates, with corrections for the beam center. Pixel binning was carried out with bounded box methodology. Fiber orientation is calculated based on the Degree of Preferred Orientation (DPO), Rulands misorientation parameters (B_ϕ_, L_f_), Herman's order parameter (*f*), and Fourier transform fitting function (FT_fit: a_1_/a_0_)^[^ ^18^, ^45^, ^46^, ^47^ ^]^. The range for orientation analysis: SAXS: 0.3 < q < 1.0 nm^−1^, WAXS: 17 < q < 23 nm^−1^.^[^ ^25^ ^‐28]^

(3) $$DPO\;=\frac{Area\;under\;peaks,\;I\left(\phi \right)}{Total\;area\;under\;curve}\;\;$$

(4) $$<\cos^{2}\phi \;{>}_{hkl}=\frac{\int_{0}^{2\pi }I\left(\phi \right)\cos^{2}\phi \sin\phi \;d\phi }{\int_{0}^{2\pi }I\left(\phi \right)\sin\phi \;d\phi }\;\;$$

(5) $$<\cos^{2}\gamma >\;=\;1-2<\cos^{2}\phi >$$

(6) $$f\;\;\left(Herman^{\prime }s\;parameter\right)=\frac{3<\cos^{2}\gamma >\;-\;1}{2}$$

(7) $$B_{obs}=\frac{1}{L_{f}*q}\;+B_{\phi }$$

(8) $$I\left(n_{\theta }\right)\approx a_{0}+a_{1}\;\cos\left(\frac{2\pi n_{\theta }}{N_{\theta }}-\theta_{s}\right)$$

### Bacterial Culture

The present work used E. coli for antibacterial performance tests. In this study, bacteria were cultured in LB broth medium in an incubator for 12 h at 37 °C. All bacteria were washed with sterilized water once before usage. The bacteria concentration was measured by OD600 and colony formation unit (CFU) on LB agar plates for bacteria loading and survival rate testing, respectively.

### The Antibacterial Performance of Aerogels

The aerogels were sterilized by UV light for half an hour. They were immersed in the *E. coli* bacteria suspension (10 mL, OD600 = 0.3, ≈4.5×10^6^ cell mL^−1^). The bacteria suspension in the tubes was shaken on a shaker (200 rpm, 37 °C). At 1, 2, 3, and 4 h, 100 µL bacteria suspension was extracted from the tubes and spread onto the LB agar plates. After that, the loaded plates were cultured in the incubator. Data were collected from three replicate tests for each of the experiments described here. The bacteria were plated in triplicate for each test, resulting in nine data points for each experiment, and their average value was used as the bacterial concentration. The survival rate of the bacteria was calculated as follows:

(9) $$\mathrm{SR}\;=\frac{N_{1}}{N_{0}}\;$$

where N_1_ was the colony number of the survived bacteria after contact with hydrogels of different times. N_0_ was the number of bacteria extracted from the suspension after the hydrogel was put into the bacteria suspension immediately.

For the fluorescence microscopy evaluation, bacteria were washed in sterilized water and stained with NucBeacon Green, following the instructions provided in the ViaQuant Viability/Cytotoxicity kit for bacteria cells (Cat. A180, GeneCopoeia, Inc.). The stained bacteria were imaged using fluorescence microscopy (Eclipse Ti‐E inverted microscope, Nikon, Zurich, Switzerland). In the fluorescence images, green fluorescence indicates live bacteria.

### Synchrotron X‐Ray Tomographic Microscopy

Aerogel monoliths were cut and tested by micro/nano computed tomography. Imaging was performed at the TOMCAT beamline of the Swiss Light Source, situated on a 2.9 T bending magnet and equipped with a multi‐layer monochromator. X‐ray images were acquired at 16 keV and a propagation distance of 25 mm. The X‐ray indirect detector comprised an LSO: Tb 5.8 µm scintillator, a 40× optical objective, and an sCMOS pco.EDGE camera (6.5 µm pixel size, 2560 × 2160 pixels), resulting in an effective pixel size of 0.1625 µm. During the continuous tomography scan, 1500 projections were collected over 180° with an exposure time of 500 ms per projection and two series of 50 flats and 20 dark projections. The data were reconstructed using the gridrec algorithm with and without prior phase‐retrieval (Paganin, delta = 6.3e^‐6^, beta = 3.6e^‐8^ with unsharp mask of 0.3 width and sigma = 1). Further segmentation and visualization were performed using a module called ImportGeo‐Vol in GeoDict (Math2Market).

In vitro evaluation of cell attachment, viability, and metabolic activity in the presence of the cellulose scaffolds – hydrogels: We evaluated the cell viability and metabolic activity in the presence of the cellulose scaffolds (10×10×1 mm^3^) using NIH/3T3 fibroblasts (CRL‐1658, ATCC). According to ISO 10993–5 standard, seven cell lines of fibroblasts of mice, hamster, and human origin are supported to assess the in vitro cytotoxicity of medical devices.^[^ ^48^ ^]^ Although every cell line has its specific sensitivity to chemicals, a good correlation between the cytotoxicity in 3T3 mouse embryonic fibroblasts and in primary or derived human cell lines have been observed in literature, showing similar^[^ ^49^, ^50^ ^]^ or even higher^[^ ^51^ ^]^ sensitivity to chemicals with toxic potential. The cells were cultured in Dulbecco's modified Eagle medium (DMEM) and 10% fetal bovine serum, and 1% penicillin‐streptomycin until reaching 80% confluency. Then, the cells were passaged using 0.25% trypsin‐EDTA and centrifuged at 500 g for 5 min to derive the cell pellet. The pellet was re‐suspended in fresh medium at a concentration of 2×10^5^ cells mL^−1^. The aerogel constructs were placed within non‐culture‐treated 12‐well plates (n = 3 per group) for viability assessments. Next, 1 mL of the cell suspension was added on top (Day 0), and the cells were allowed to attach over the aerogel well plates overnight. On days 1 and 3, the wells were evaluated for viability using the Live/Dead cytotoxicity kit (L3224, Thermo Fisher Scientific).

Metabolic activity assessment was performed using an MTT assay similar to previous work.^[^ ^52^ ^]^ Briefly, 10000 cells were seeded into a transwell (Day 0) within a 24‐well plate, and 120 µL of the medium was added on top. The scaffolds were cut to 5×5 mm^2^ and placed underneath the transwells, and 600 µL of medium was added. Every second day (i.e., days 1, 3, 5, and 7), the Transwell inserts with the cells were extracted and then transferred to empty wells, followed by the removal of the existing medium and the addition of 120 µL of MTT assay reagent (prepared using the manufacturer's protocol, 11 465 007 001, Millipore Sigma, St Louis, MO, USA). The cells were then incubated for 4 h at 37 °C. The MTT assay was then collected over the Transwell, followed by washing the cells with PBS and replacing them with a fresh medium. The Transwells were inserted back into the original wells with fresh 600 µL medium. The absorbance of the supernatant MTT reagent (100 µL) was measured at 570 nm.

In vitro evaluation of cell attachment, viability, and metabolic activity in the presence of the cellulose scaffolds – aerogels: The resazurin (Sigma–Aldrich) conversion into resorufin by metabolically active cells was employed to assess cell viability of aerogels. Mouse embryo fibroblasts (NIH/3T3) were seeded in 24‐well plates (2000 cells cm^−2^) with 600 µL of Dulbecco's Modified Eagle's Medium growth media supplemented with 10% bovine calf serum, penicillin 100 U mL^−1^ and streptomycin 100 g mL^−1^. Scaffolds of 10 mg were UV‐sterilized for 30 min and then placed in culture inserts in contact with cells and incubated at 37 °C in an atmosphere with 5% CO_2_. After 24 and 72 h, 100 µL of 44 µM resazurin was added into each well, and its extinction was measured at 544 nm after an incubation of 3 h under the same conditions with an ELISA reader. All experiments were carried out in triplicate, and cell control without scaffolds was employed.

## Conflict of Interest

The authors declare no conflict of interest.

## Supporting information

Supporting Information

Supplemental Movie 1

Supplemental Movie 2

Supplemental Movie 3

Supplemental Movie 4

## Data Availability

The data that support the findings of this study are available from the corresponding author upon reasonable request.
