# Supplementary material for: Additive Manufacturing of Nanocellulose Aerogels with Structure‐Oriented Thermal, Mechanical, and Biological Properties
Source: Adv Sci (Weinh). 2024 Mar 13;11(24):2307921. doi: 10.1002/advs.202307921 (PMC11580277; doi:10.1002/advs.202307921)
Supplement: Supplementary file 1 — Supporting Information [file ADVS-11-2307921-s004.pdf]

ADVANCED SCIENCE
Open Access

Supporting Information

for *Adv. Sci.*, DOI 10.1002/advs.202307921

Additive Manufacturing of Nanocellulose Aerogels with Structure-Oriented Thermal, Mechanical, and Biological Properties

Deeptanshu Sivaraman, Yannick Nagel, Gilberto Siqueira, Parth Chansoria, Jonathan Avaro, Antonia Neels, Gustav Nyström, Zhaoxia Sun, Jing Wang, Zhengyuan Pan, Ana Iglesias-Mejuto, Inés Ardao, Carlos A. García-González, Mengmeng Li, Tingting Wu, Marco Lattuada, Wim J. Malfait* and Shanyu Zhao**

Supporting Information

Additive manufacturing of nanocellulose aerogels with structure-oriented thermal, mechanical, and biological properties

Deeptanshu Sivaraman, Yannick Nagel, Gilberto Siqueira, Parth Chansoria, Jonathan Avaro, Antonia Neels, Gustav Nyström, Zhaoxia Sun, Jing Wang, Zhengyuan Pan, Ana Iglesias-Mejuto, Inés Ardao, Carlos A. García-González, Mengmeng Li, Tingting Wu, Marco Lattuada , Wim J. Malfait*, Shanyu Zhao**

This supporting file includes the following:

Figure S1 to S9

Table S1

Other Supplementary Materials for this manuscript include the following:

Movies S1 to S4

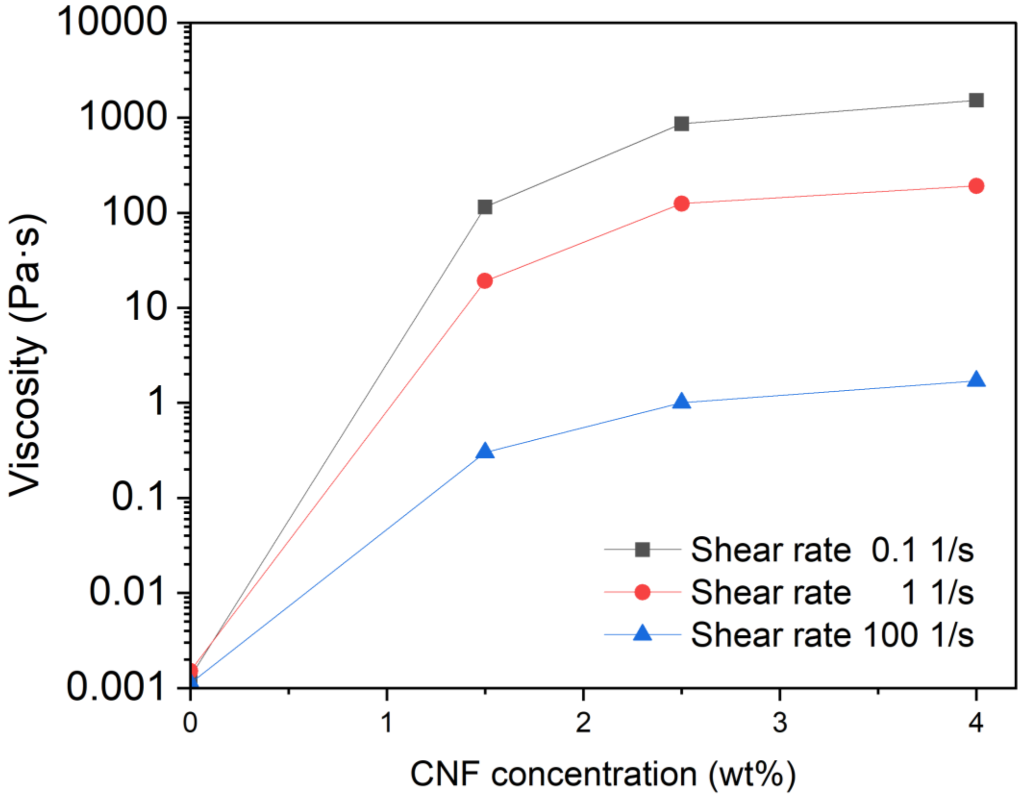

Supplementary Figure S1. Viscosity of the CNF sols with different CNF concentrations and performed at different shear rates

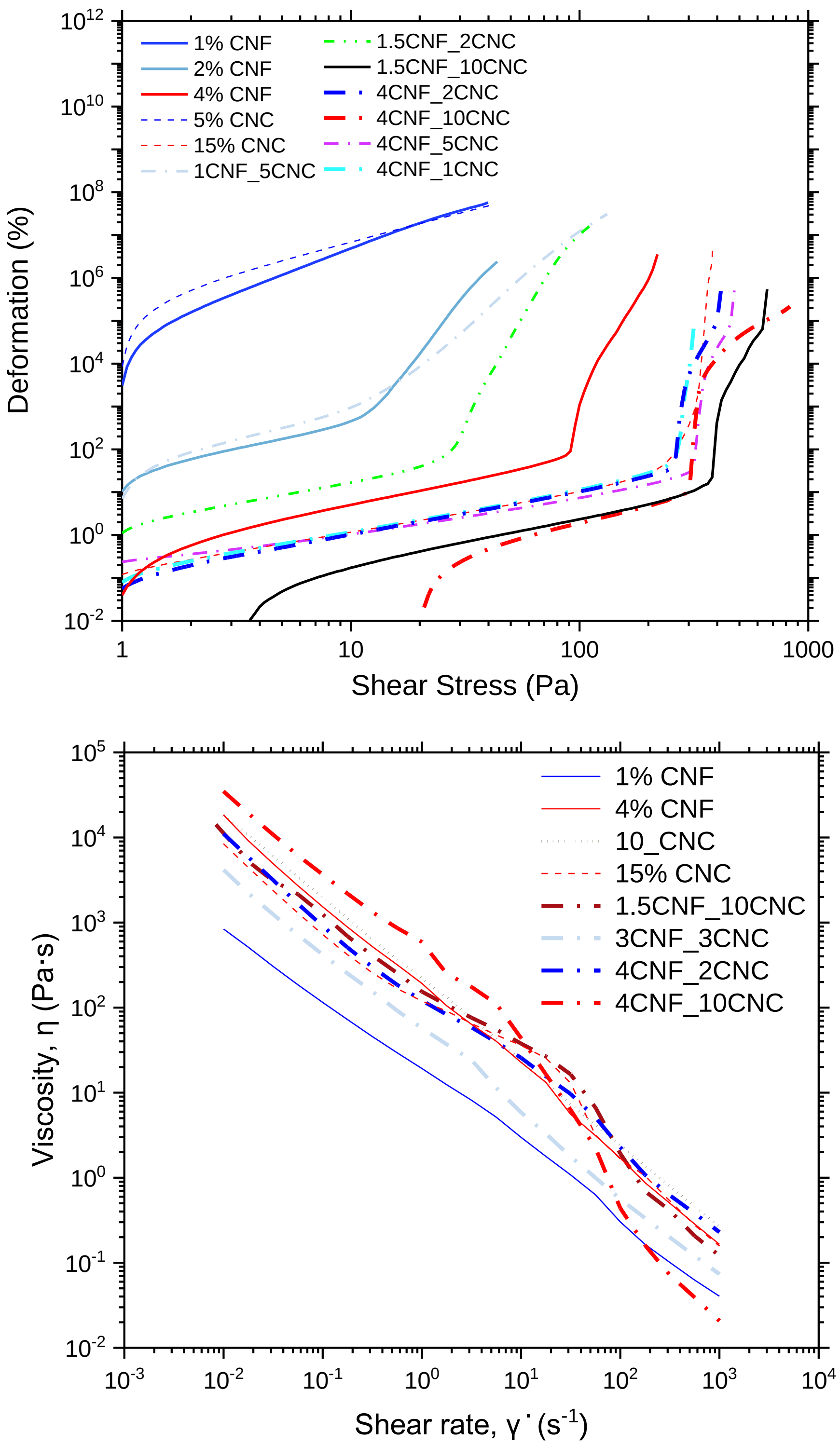

Supplementary Figure S2. Rheological data for all the inks studied in the article: **a.** Amplitude sweep of inks, and **b.** Shear-thinning behavior.

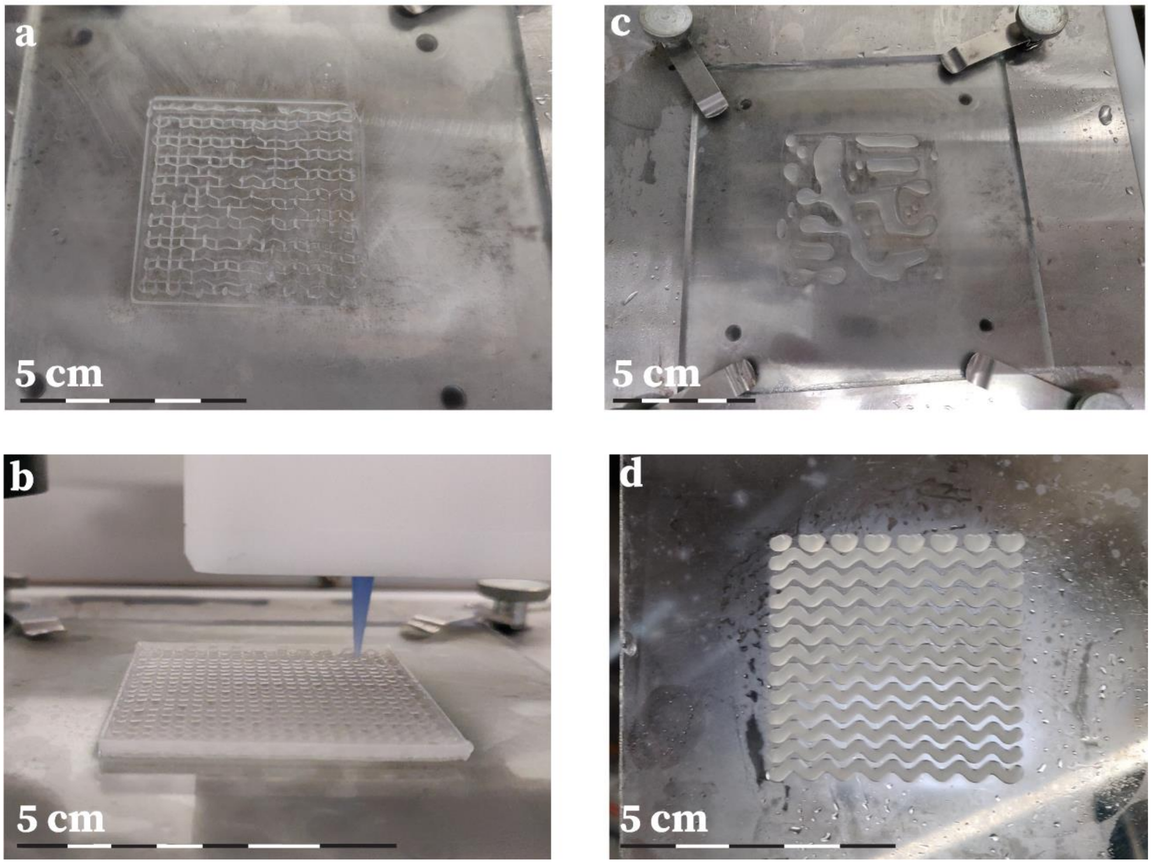

Supplementary Figure S3. a. Poor printing parameters (speed and pressure) during 4CNF_10CNC printing; **b.** Optimum printing parameters for 4CNF_10CNC; **c.** 1.5CNF_2CNC 'printing' showing poor rheological performance; **d.** 4CNF_2CNC printing of complex wave-like pattern.

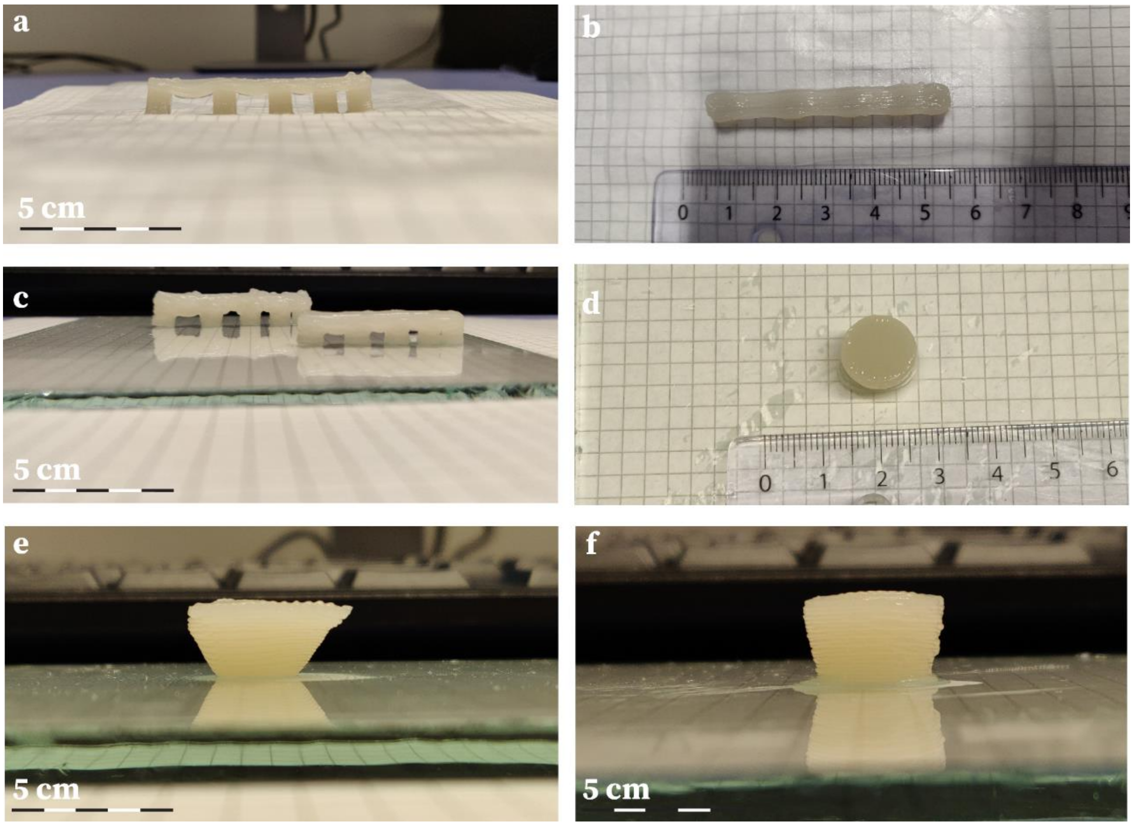

Supplementary Figure S4. Bridging test for 4CNF_10CNC **a.** side-view; **b.** top-view; **c.** Bridging test for 1.5_CNF_10CNC, side-view; Overhanging tests for 4CNF_2CNC, **d.** 90° angle **e.** 60° angle **f.** 75° angle

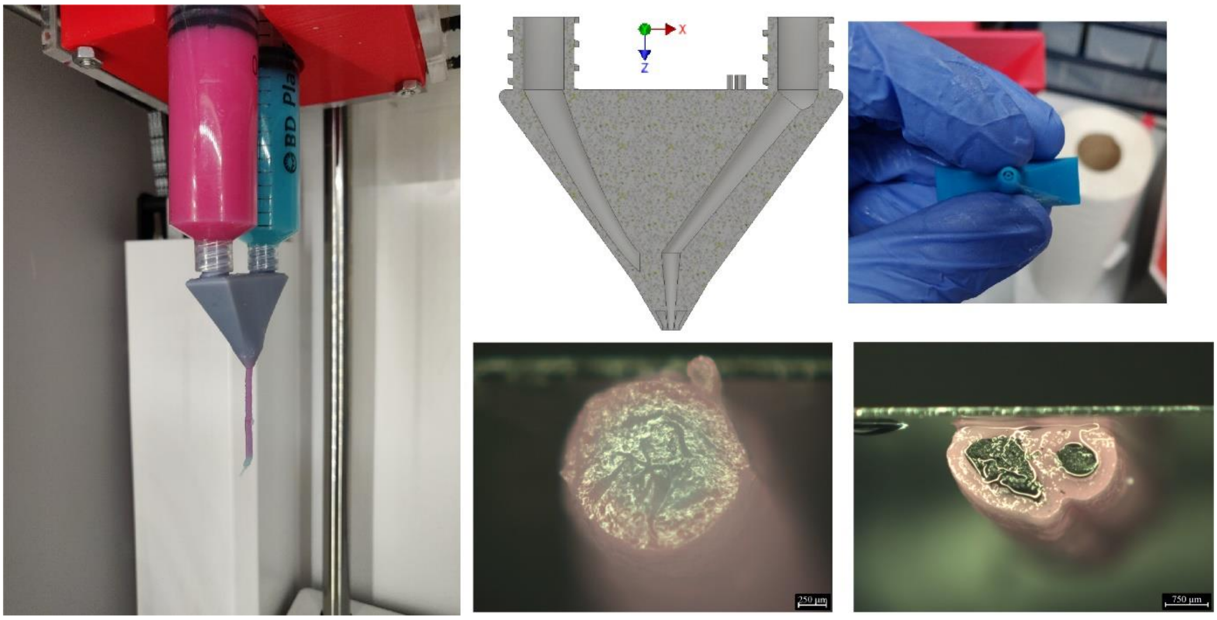

Supplementary Figure S5. Different stages of printing two cellulose inks simultaneously, with design possible for hollow tubes that hold shape fidelity after printing.

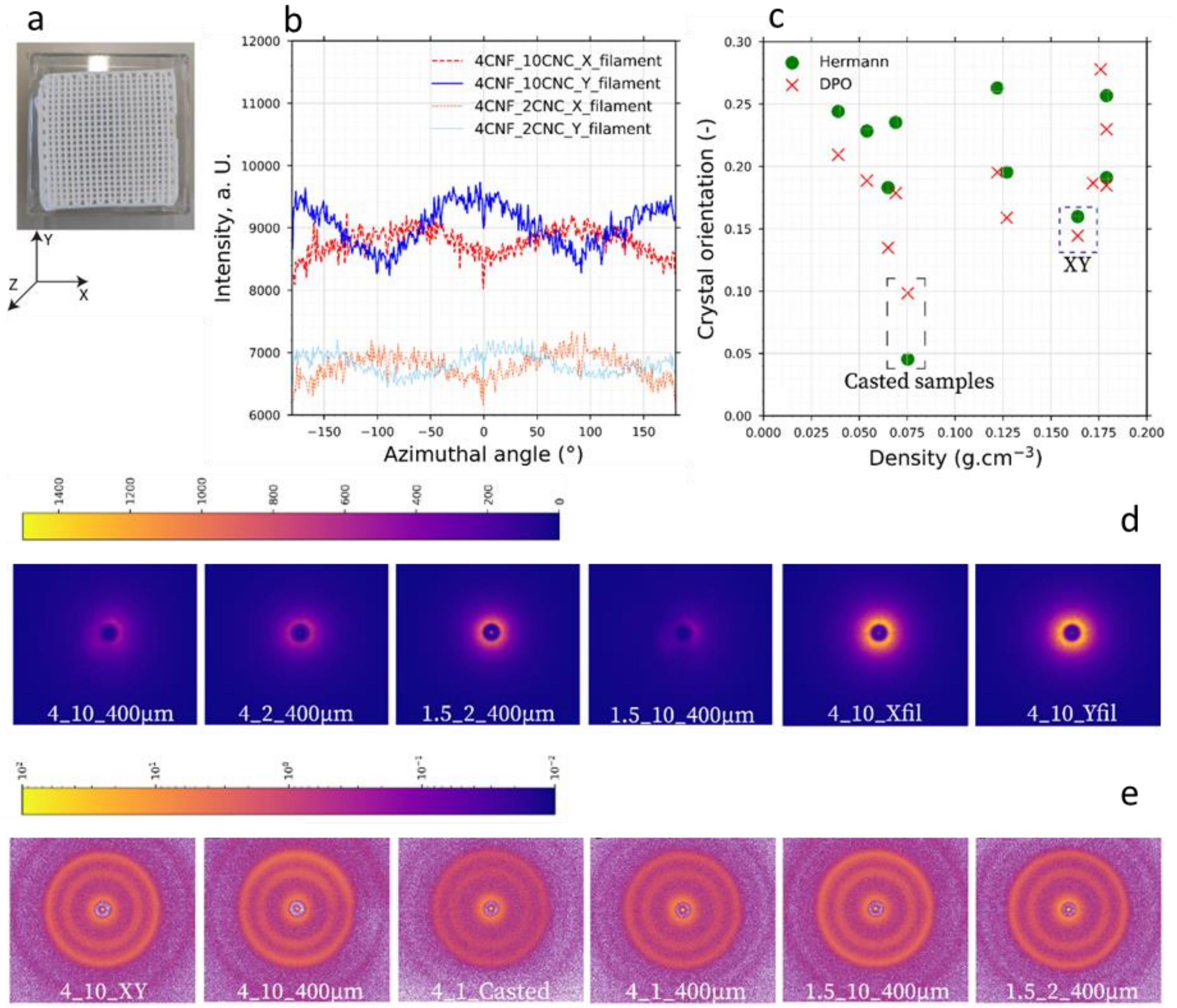

Supplementary Figure S6. a. Grid pattern sample, **b.** Radial integration of SAXS profiles of two different CNC concentrations, with X (dashed lines) and Y (solid lines) filament showing a phase difference of 90° for monoliths; **c.** Crystal orientation analysis using Herman's order parameter and DPO; **d.** showing the SAXS profiles for each of the different formulations investigated with color bar, **e.** WAXS orientation profiles for different cellulose formulations, with color bar in log-scale. (azimuthal profiles were obtained via the radial integration of q-values between $17 < q < 23$ nm^{-1})

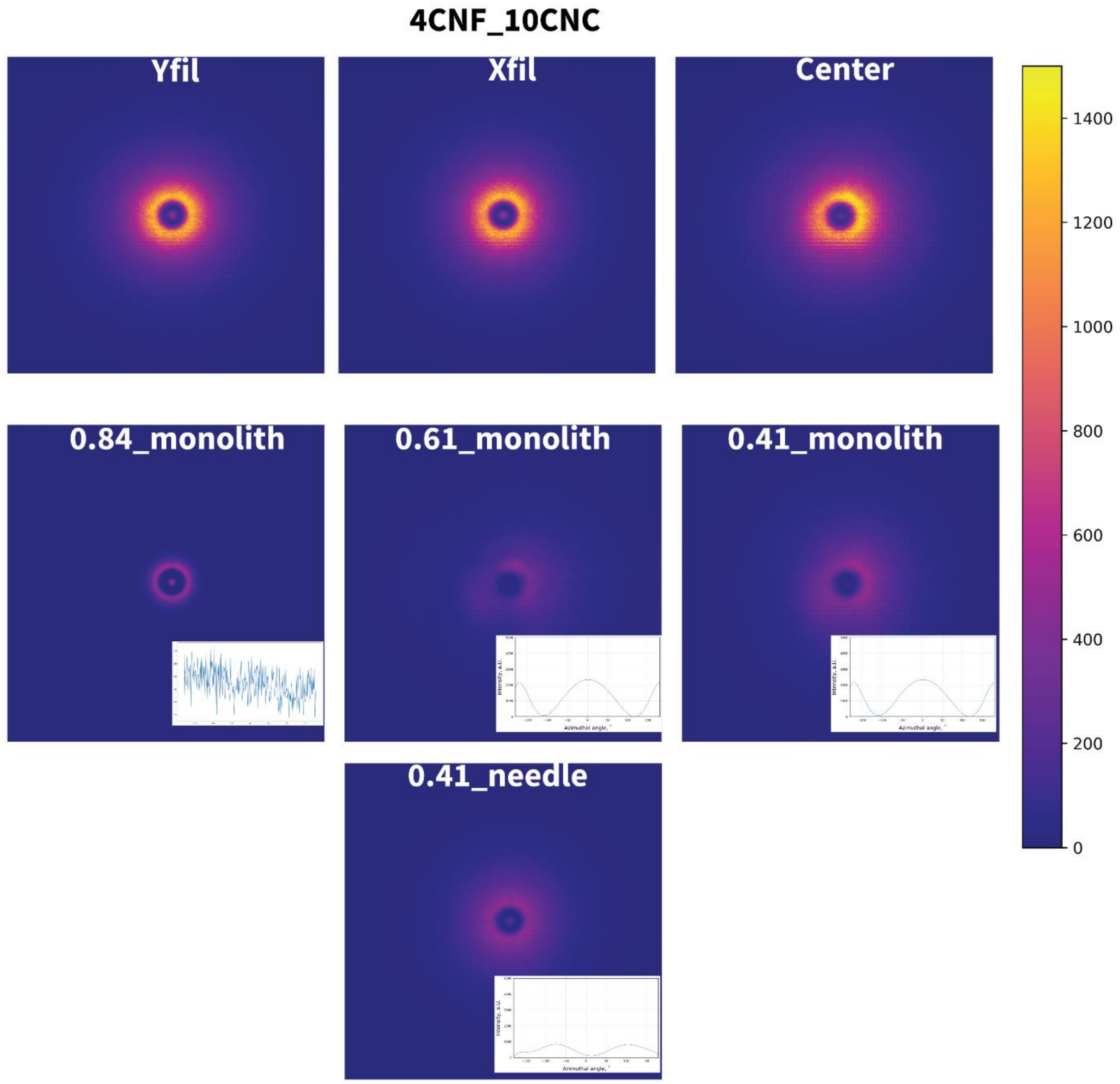

Supplementary Figure S7. SAXS profiles, with a radial integral in the inset to show the alignment of the chosen nozzle size. Concentration was set at 4CNF_10CNC.

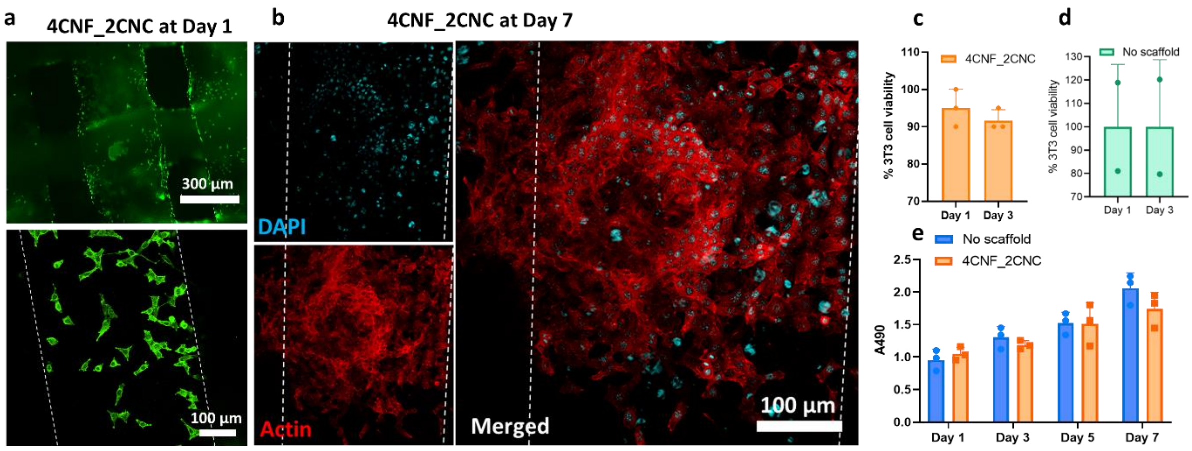

Supplementary Figure S8. a, b. NIH/3T3 fibroblasts encapsulated in 4CNF_2CNC hydrogel formations after (a) 1 day and (b) 7 days, and **c.** In culture, NIH/3T3 cell viability on 4CNF_2CNC fresh hydrogels was high after 1 and 3 days (> 90%). **d.** Control of the cell viability test with no scaffold, **e.** Cellular metabolic activity (determined from the MTT assay) of cells cultured in the presence of the fresh cellulose hydrogel was not significantly different from the controls (no scaffold) during 1, 3, 5, and 7 days of culture.

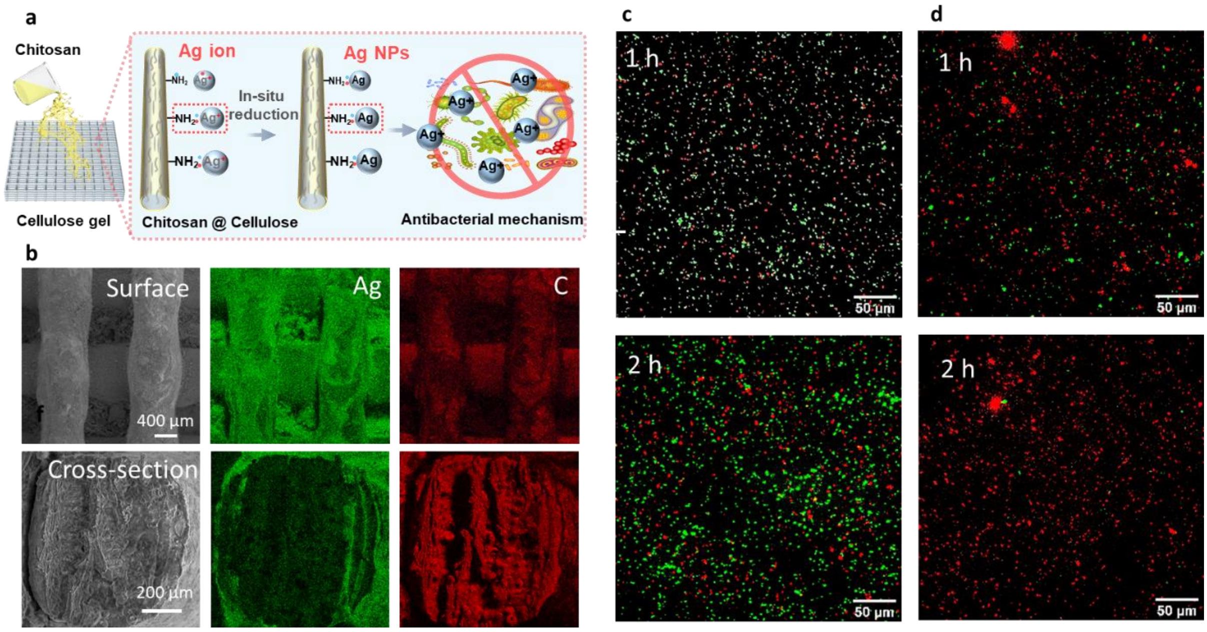

Supplementary Figure S9. a Process scheme: DIW printed cellulose objects were immersed in the chitosan solution (5 wt.%) and then loaded with silver ions; the composites were exposed to UV irradiation (95% at 254 nm) for 10 hrs to in-situ form the Ag nanoparticles [38]. **b.** SEM surface and cross-section of dried grid structures, the EDX elemental (Ag and C) mapping, and **c, d.** The fluorescence microscopy images of the NucBeacon Green stained bacteria in the (c) controlled cellulose scaffold and (d) Ag-NP loaded cellulose/chitosan scaffold after 1 and 2 hours. In the fluorescence images, green fluorescence indicates live bacteria.

Supplementary Table S1. Relevant aerogel properties of different CNF_CNC formulations

Name	Nozzle	λ	Envelope Density	BET SA	V_{pore}, calc	D_{pore}, calc	V_{pore}, BJH	D_{pore} BJH	Mesopore
%CNF_%CNC	mm	mW m^{-1} K^{-1}	g cm^{-3}	m^2 g^{-1}	cm^3 g^{-1}	nm	cm^3 g^{-1}	nm	%
**1.5_2**	0.41	30.1	0.038	367	25.69	161.66	1.88	23	7.32
**2_10**	0.41	48.3	0.118	292	7.85	62.08	1.1	--	14.01
**3_10**	0.41	51.4	0.145	275	6.27	52.67	1.64	--	26.15
**4_2**	0.41	27.1	0.067	379	14.30	87.14	1.12	15	7.83
**1.5_10**	0.4	45.7	0.115	300	8.07	62.13	1.2	18	14.87
**1.5_10**	0.4needle	43.5	0.12	321	7.71	55.46	2	27	25.95
**4_10**	0.4	56.4	0.175	304	5.09	38.66	1.42	21	27.90
**1.5_10**	0.84	46.5	0.144	235.0	6.32	62.10	1.56	--	24.6
**4_2**	0.84	26.8	0.064	335.0	15.00	103.41	1.3	18	8.67
**4_1**	0.84	24.4	0.055	414.0	17.56	97.94	1.2	15	6.83
**3_10**	0.84	54.5	0.14	236.0	6.52	63.78	1.45	26	22.25
**4_10**	0.84	56.1	0.165	310.0	5.44	40.49	2.1	29	38.63
**2_10**	0.84	49.5	0.122	295	7.57	59.28	1.01	17	13.34
**F_2**	0.4 needle	27.5	0.06	410	16.04	90.36	1.77	--	11.03
**4_10**	0.4 needle	53.6	0.185	242	4.78	45.62	0.99	20	20.71
**4CNF**	Casted	28.5	0.035	466	27.95	138.50	2.25	37	8.05
**10% CNC**	0.41	38.2	0.175	345	5.09	34.07	1.75	24	34.39
**15% CNC**	0.41	51.2	0.25	332	3.38	23.48	1.46	19	43.26

Movie S1.
Bridging test with ink 4CNF_10CNC through 410 µm conical nozzle.

Movie S2.
Print longitudinal orientation with ink 4CNF_10CNC through an 840 µm conical nozzle.

Movie S3.
Precisely control the pattern and geometry from ink 4CNF_10CNC through a 410 µm metallic nozzle.

Movie S4.
Print gel grids with ink 4CNF_10CNC through a 410 µm metallic nozzle.
